# Cross-cultural adaptation and validation of the German version of the Birth Satisfaction Scale-Revised (BSS-R)

**DOI:** 10.1186/s12889-025-25563-2

**Published:** 2025-11-22

**Authors:** Lara Seefeld, Amera Mojahed, Colin R. Martin, Caroline J. Hollins Martin, Julia Schellong, Susan Garthus-Niegel

**Affiliations:** 1 Department of Psychology and Neuroscience, Dalhousie University, Halifax, Canada; 2 Institute and Policlinic of Occupational and Social Medicine, Faculty of Medicine, Technische Universität Dresden, Dresden, Germany; 3 Department of Psychology, Faculty of Social Sciences, Vytautas Magnus University, Kaunas, Lithuania; 4 Institute for Health and Wellbeing, University of Suffolk, Ipswich, UK; 5 School of Health and Social Care, Edinburgh Napier University, Edinburgh, UK; 6 Department of Psychotherapy and Psychosomatic Medicine, Faculty of Medicine, Technische Universität Dresden, Dresden, Germany; 7 Institute for Systems Medicine (ISM), Faculty of Human Medicine, MSH Medical School Hamburg, Hamburg, Germany; 8 Department of Childhood and Families, Norwegian Institute of Public Health, Oslo, Norway

**Keywords:** Birth experience, Birth satisfaction, Validation, Postpartum

## Abstract

**Background:**

Up to one third of women are dissatisfied with their birth experience. A negative birth experience can have detrimental outcomes, making an early detection of dissatisfaction with the birth experience very important. The Birth Satisfaction Scale-Revised (BSS-R) is a multi-dimensional measure of birth satisfaction, which has been translated into several languages. The current study aimed to translate and validate a German version of the BSS-R (DE-BSS-R).

**Methods:**

A total of 3747 German women, who were participating in the cross-sectional study INVITE, completed the DE-BSS-R 3–4 months postpartum. The factor structure of the DE-BSS-R was tested using confirmatory factor analysis. Moreover, internal consistency as well as known-groups discriminant, divergent, convergent, and predictive validity were evaluated.

**Results:**

Both the tri-dimensional and the bi-factor measurement model of the original BSS-R showed excellent fit to the data. Internal consistency was acceptable for the total score and the subscale *Women’s personal attributes*, but just below the recommended cut-off for the subscales *Stress experienced during labour* and *Quality of care*. However, Cronbach’s alpha did not differ significantly from the acceptable alpha values of the original BSS-R. Women with a non-instrumental vaginal birth had significantly higher birth satisfaction than women with an instrumental birth (instrumental vaginal, caesarean section). The DE-BSS-R showed good divergent, convergent, and predictive validity.

**Conclusions:**

Overall, the German cross-cultural adaptation of the BSS-R showed excellent psychometric properties. Both the total score and the three subscale scores are valid to quickly measure German women’s satisfaction with birth in research and clinical practice.

**Supplementary Information:**

The online version contains supplementary material available at 10.1186/s12889-025-25563-2.

## Background

The birth of a child is a major life event and life-changing experience for parents. Using an inclusive, women-centred approach, members of the COST Action CA18211 (https://www.ca18211.eu/) on childbirth-related trauma have defined a positive birth experience as “a woman’s experience of interactions and events directly related to childbirth that made her feel supported, in control, safe, and respected” as well as having the ability to “make women feel joy, confident, and/or accomplished” [1]. However, a systematic review and meta-analysis found that up to 34% of women worldwide experience the birth of their child as negative [2]. A negative birth experience is often associated with a long duration of labour [3], a birth mode which is characterized by high interventions (e.g., instrumental vaginal birth, unplanned caesarean section) [3, 4], a mismatch between the mother’s expectations and actual experience of birth [5], low maternal involvement in decision making during birth [6], and support from the midwife and/or birth partner being perceived as insufficient by women [7]. In addition, obstetric violence (e.g., disrespectful communication, discrimination, psychological, or even physical and sexual violence) by staff in the delivery room is increasingly discussed as an influential adverse factor for women’s birth experience [8].

The detrimental outcomes of a negative birth experience have been investigated by many researchers worldwide. These studies show that dissatisfaction with the birth experience may lead to a feeling of disempowerment [9], but also predicts (long-term) symptoms of postpartum depression and childbirth-related posttraumatic stress disorder (CB-PTSD) [10–12]. These disorders can have far-reaching consequences for the mother as well as the infant and the whole family [13, 14], therefore, early detection of mothers with a negative birth experience and appropriate interventions, such as a conversation with an empathic listener soon after birth [15] or supportive counselling [16], is of utmost importance to prevent the development of maternal postpartum mental disorders [17].

Due to high reliability, low costs, and easy administration, validated self-report questionnaires should be used to assess women’s birth satisfaction. The International Consortium for Health Outcomes Measurement (ICHOM) has recommended the Birth Satisfaction Scale-Revised (BSS-R) [18] as the tool of choice for assessing satisfaction with the birth experience around the globe. The questionnaire consists of 10 items on three subscales: [1] quality of care provision [2], women’s personal attributes, and [3] stress experienced during labour and has been translated into several languages (e.g., Croatian, Dutch, Spanish, Swedish, and Urdu) [19–23]. A German-language translation of the BSS-R has been developed and tested for cultural and linguistic comprehensibility among a group of five postpartum women [24]. This version, the Ger-BSS-R, has recently been validated in a sample of German women with severe health conditions affecting pregnancy [25], showing adequate fit to the expected measurement models and acceptable internal consistency for the total score and women’s personal attributes subscale but inadequate internal consistency of the other two subscales.

The validation of a German version of the BSS-R in a community sample is still needed. Therefore, the current study aimed to validate the German translation of the BSS-R in a large community sample of German postpartum women by empirically testing the following predictions, which were based on previous validations of the BSS-R in other languages:

- The tri-dimensional measurement model of the original BSS-R will demonstrate good model fit.
- The bi-factor model of the German BSS-R will demonstrate acceptable model fit.
- The internal consistency of the German BSS-R total scale and subscales will be acceptable.
- The German BSS-R will show good known-groups discriminant validity when using birth mode as a between-groups factor.
- The German BSS-R will demonstrate acceptable divergent validity with non-significant correlations with women’s age.
- The German BSS-R will demonstrate good convergent validity with significant negative correlations with women’s CB-PTSD symptoms.
- The German BSS-R will demonstrate good predictive validity with significant negative regression coefficients for women’s CB-PTSD and depression symptoms.

We also explored whether the German BSS-R would equally measure birth experience in women with lower and higher levels of education.

## Methods

### Design and sample

This study is based on version 4 of the quality-assured data files of the cross-sectional study INVITE (INtimate partner VIolence Treatment prEferences) [26], which investigates the treatment and counselling preferences of postpartum women from Dresden, Germany. Women were mostly recruited by trained student assistants who approached women at midwife antenatal appointments at the hospital, on the maternity ward, or in free-standing, midwifery-led birth centres between November 2020 and October 2023. During the COVID-19-related lockdowns, the student assistants were not allowed in all recruitment sites and therefore, midwives at the respective hospitals gave out the study information material to interested women. In one hospital, student assistants presented the study and gave out information material at the regular birth information events, while midwives also distributed the study material at the antenatal appointments. Additionally, employees of the Dresden Youth Welfare Office distributed the study material at their Welcome Visits, which are offered to all families with newborns in the city of Dresden. All women ≥ 16 years with sufficient German skills were included in the current study. They were invited to participate in a one-hour long telephone interview, and the interviews were conducted between January 2021 and February 2024. We aimed to conduct the interviews three to four months postpartum, however, due to preterm births, scheduling issues, and difficulties contacting some women, some women completed the interview before or after this time frame. After the interview, women received 20€ as monetary compensation.

### Birth Satisfaction Scale-Revised (BSS-R) and its cross-cultural adaptation into German

The BSS-R [18] is a validated 10-item questionnaire, which examines the satisfaction with the birth experience. Answers are scored on a five-point Likert-scale ranging from *strongly disagree* (0) to *strongly agree* [4] and all items are summed to form a total score between 0 and 40, where higher scores indicate greater birth satisfaction. Items 2, 4, 7, and 8 are reverse coded. The scale encompasses three distinct but correlated subscales: [1] quality of care provision (QC) [2], women’s personal attributes (WA), and [3] stress experienced during labour (SE). The QC subscale score is formed by summing items 3, 5, 6, and 10. The WA subscale score is formed by summing items 4 and 8, and lastly, the SE subscale score is formed by summing items 1, 2, 7, and 9.

The BSS-R was translated using the back-translation method of the World Health Organization in November 2020, before launching the INVITE study. We did not know of the alternative German translation of the BSS-R by Hartmann et al. [24], as this version had not been published yet, which is why we did not use it in our study. Since the version by Hartmann et al. is called ‘Ger-BSS-R’, we have chosen to name our version ‘DE-BSS-R’ to facilitate the differentiation of both versions.

The original English-language (UK) version was first translated into German by the first author of this paper, who is a psychologist specialized in perinatal mental health. Her mother tongue is German, and she is fluent in English. The translation was discussed in an online meeting with an expert panel consisting of the other research team members (1 psychologist, 2 psychology master’s students, 1 psychiatrist, 1 public health researcher) and amended accordingly. In a next step, this version A was back-translated into English by two independent people (one public health researcher (JH), one German and English teacher (LW)) whose mother tongue is English, and who are fluent in German and were unfamiliar with the BSS-R. The three versions were compared and discussed with the authors of the original BSS-R [18], which resulted in no changes compared to version A. Subsequently, during a telephone interview, the DE-BSS-R was tested on two mothers with small children, who had no difficulty understanding and answering all items. As they had no suggestions for improvements, no changes were made to the DE-BSS-R.

Only a few words of the original BSS-R were difficult to translate into German. The greatest challenge was translating the word ‘labour’ in items 2, 6, and 9, because there is no direct equivalent in German. The word closest to ‘labour’ (‘Wehen’) also means ‘contractions’ and could therefore have been confusing to women. After deliberating with the authors of the original BSS-R, we decided to use ‘labour and birth’ in items 2 and 6, because these items refer to the whole birth process from the first contraction to the birth of the placenta. In item 9, only ‘labour’ was used because this refers more specifically to the first stages of labour before the birth of the baby. The alternative German translation by Hartmann et al. [24] used the German translation for ‘birth’ in all items, irrespective of whether the original item said ‘labour’, ‘birth’, or ‘labour and birth’. Overall, this translation is similar to the DE-BSS-R, with our version being closer to the wording of the original UK version. The final translation of all items of the DE-BSS-R can be found in the supplemental material (see Table S1).

### Data collection

Data collection and management was facilitated using Research Electronic Data Capture (REDCap), a secure, web-based application for data capture as part of research studies, hosted at the “Koordinierungszentrum für Klinische Studien” at the Faculty of Medicine of the Technische Universität Dresden [60, 61].

#### Edinburgh Postnatal Depression Scale (EPDS)

The EPDS [27] is a 10-item questionnaire, which screens for symptoms of postpartum depression during the last week (e.g., “I have felt sad or miserable.”). Answers are scored on a 4-point Likert-type scale, ranging from 0 to 3, with a total score between 0 and 30 and higher scores indicating more depressive symptoms. The German version of the EPDS has been validated and is widely used in research and clinical practice [28].

#### City Birth Trauma Scale (City BiTS)

The City BiTS [29] is a 29-item questionnaire, which examines symptoms of CB-PTSD according to the criteria of the Diagnostic and Statistical Manual of Mental Disorders 5th Edition (DSM-5). Most questions are answered on a 4-point Likert scale (*not at all*,* once*,* 2–4 times*,* 5 or more times*), the total score ranges between 0 and 60, and higher scores indicate more CB-PTSD symptoms. Women are asked about the four symptom clusters re-experiencing, avoidance, negative cognitions and mood, and hyperarousal, which are divided into two subscales: birth-related (e.g., “Flashbacks to the birth and/or reliving the experience”) and general posttraumatic stress disorder (PTSD) symptoms (e.g., “Feeling detached from other people“). The German version of the City BiTS has been validated with good psychometric properties [30].

### Data analysis

Apart from the confirmatory factor analyses (CFA), which were conducted in M*plus* version 8 [31], all analyses were computed using SPSS version 29. A two-sided alpha significance level of 0.05 was used for all significance tests. If women were missing less than 20% of the responses of a questionnaire, their missing response to single items was replaced by their mean score of answered items. Full information maximum likelihood estimation was used to handle missing data for the CFA, but listwise deletion was employed for all other analyses.

### Confirmatory factor analysis

In preparation for CFA, multivariate outliers were detected using Mahalanobis distance and excluded from the sample. Next, multivariate normality was checked by assessing skew and kurtosis for each item of the DE-BSS-R. Data were considered non-normally distributed if skew ≥ 2 and kurtosis ≥ 7 [32, 33]. Additionally, Pearson correlations were computed for the total score and the three subscale scores and correlations were compared to the correlations of the original UK version of the BSS-R using the approach of Diedenhofen and Musch [34].

The tri-dimensional measurement model comprising the three correlated subscales SE, WA, and QC was tested using Maximum Likelihood estimation in case of normally distributed data and robust Maximum Likelihood estimation in case of non-normally distributed data. Additionally, a bi-factor model comprising a general factor with all items and three uncorrelated factors consisting of the three BSS-R subscales was tested as this model also showed acceptable model fit in other validations [19, 22, 35]. Model fit was evaluated using the comparative fit index (CFI) [36], the root mean squared error of approximation (RMSEA) [37], and the square root mean residual (SRMR) [38]. CFI >0.90 [39], RMSEA < 0.08 [40], and SRMR < 0.08 [38] were considered a good model fit to the data.

### Multigroup CFA for educational groups

In a post-hoc test, we conducted a multigroup CFA to test measurement invariance of the DE-BSS-R across educational groups for the tri-dimensional measurement model. In Germany, students can leave school with a school leaving certificate after Year 9, Year 10, and after Year 12 or 13 (depending on the federal state), which are all considered different educational degrees and allow the student to pursue different careers (i.e., apprenticeship with Year 9 or Year 10 degrees vs. university with Year 12/13 degree). We therefore divided women into two groups for our analysis: women with 10 or less years of education vs. women with more than 10 years of education. We evaluated configural, metric, and scalar invariance sequentially. As there is evidence that the chi-square difference test is overpowered in the context of measurement invariance testing, especially for large samples such as the current one, we evaluated change in RMSEA, CFI, and SRMR to determine whether the assumptions of measurement invariance are tenable [41]. A change in RMSEA, CFI, and SRMR ≤ 0.01 indicated a non-substantive worsening of model fit [42].

#### Internal consistency

Internal consistency of the total score and the scores of the SE and QC subscales were investigated using Cronbach’s alpha, with values >0.70 demonstrating acceptable internal consistency. These alpha values were compared to the alpha values of the original UK version of the BSS-R using the method of Diedenhofen & Musch [43]. Following the approach of previous BSS-R validations, inter-item Pearson correlation (due to normally distributed items and the assumption of an interval scale) was used to evaluate the two-item WA subscale, with 0.15–0.50 demonstrating an acceptable range [44]. Moreover, McDonalds Omega (*ω*), Omega hierarchical (*ωh)*, and Omega total (*ωt)* were calculated for the total score, as *ω* has been suggested to be a better indicator for total scale internal consistency [45] and *ωh* as well as *ωt* should be reported in addition to Cronbach’s alpha of a total scale [46].

#### Known-groups discriminant validity

Following the approach of previous BSS-R validations, known-groups discriminant validity was evaluated by comparing women’s BSS-R total score and subscale scores as a function of their birth mode: non-instrumental vaginal birth (i.e., without the use of forceps or vacuum extraction) vs. instrumental birth (instrumental vaginal birth using forceps or vacuum extraction, planned caesarean section, emergency caesarean section). According to the findings of previous validations, we predicted significantly higher total scores and SE and WA subscales scores in women with a non-instrumental vaginal birth compared to instrumental birth but had no specific prediction for the QC subscale scores. In addition, we compared women based on the type of caesarean section and predicted significantly higher total and SE and WA subscale scores for women with a planned caesarean section compared to an emergency caesarean section. For all comparisons, unpaired t-tests were computed, and Cohen’s *d* was reported as the effect size, with *d* > 0.2 representing a small effect, *d* > 0.5 representing a medium effect, and *d* > 0.8 representing a large effect.

#### Divergent validity

Divergent validity was tested by computing Pearson’s correlations (due to normally distributed total and subscale scores and normally distributed age and continuous scores) between women’s DE-BSS-R total and subscale scores and age. Non-significant correlations were predicted.

#### Convergent validity

Convergent validity was tested by computing Spearman’s rho correlations (due to non-normally distributed birth-related and general PTSD subscale scores) between the DE-BSS-R total and subscale scores with the City BiTS birth-related PTSD and general PTSD subscale scores. According to previous validations [22], it was predicted that DE-BSS-R total and subscale scores will show a significant negative correlation with the birth-related and general PTSD subscale, with the latter correlation being smaller than the former.

#### Predictive validity

Predictive validity was tested by regressing the City BiTS subscale scores of birth-related and general PTSD and EPDS total scores on DE-BSS-R subscale scores in three separate regression analyses. Significant negative regression coefficients were predicted for all outcomes.

## Results

### Sample characteristics

The response rate for the current data was 45.8%, resulting in a total of *N* = 3816 telephone interviews with postpartum women. Mahalanobis distance detected 69 multivariate outliers (1.8%), which were removed from the dataset, resulting in *n* = 3747. The characteristics of the sample can be found in Table 1. The majority of women were in a partnership (97.7%), born in Germany (93.1%), had more than 10 years of education (73.4%), and moderate to high income.

**Table 1:** Sample characteristics ^a^

Characteristics	M (SD)	Range
Maternal age (in years)	32.9 ± 4.7	16.8–54.0
Age of newborn (in months)	13.2 ± 3.0	1.6–52.6

	***n***	**%**
Partnership status		
Partner	3660	97.7
No Partner	87	2.3
Country of birth		
Germany	3490	93.1
Other country	257	6.9
Education		
≤ 10 years	997	26.6
>10 years	2747	73.4
Income ^b^		
< 1,250€ – 1,749€	247	6.6
1,750€ – 2,999€	694	18.6
3,000€ – 4,999€	1,903	60.0
>5,000€	888	23.8
Parity		
Primiparous	1958	52.3
Multiparous	1789	47.7
Birth mode		
Non-instrumental vaginal birth	2764	73.8
Instrumental vaginal birth ^c^	237	6.3
Planned cesarean section	458	12.2
Emergency cesarean section	287	7.7

^a^ *n* varies slightly due to missing data, ^b^ net income per household and month, ^c^ forceps or vacuum extraction

### Confirmatory factor analysis

**Fig. 1:**
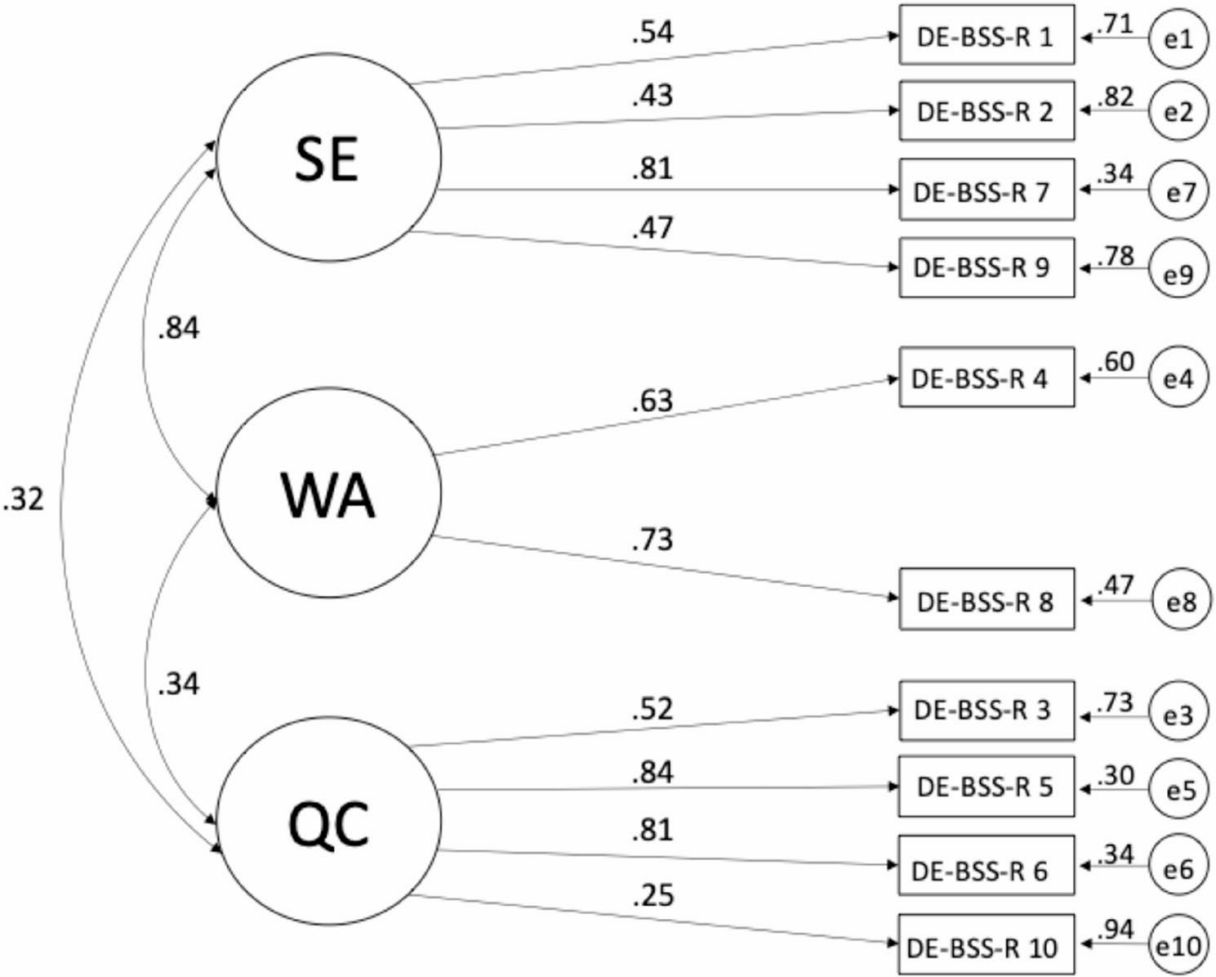
Standardized factor loadings of the tri-dimensional measurement model of the DE-BSS-R

**Fig. 2:**
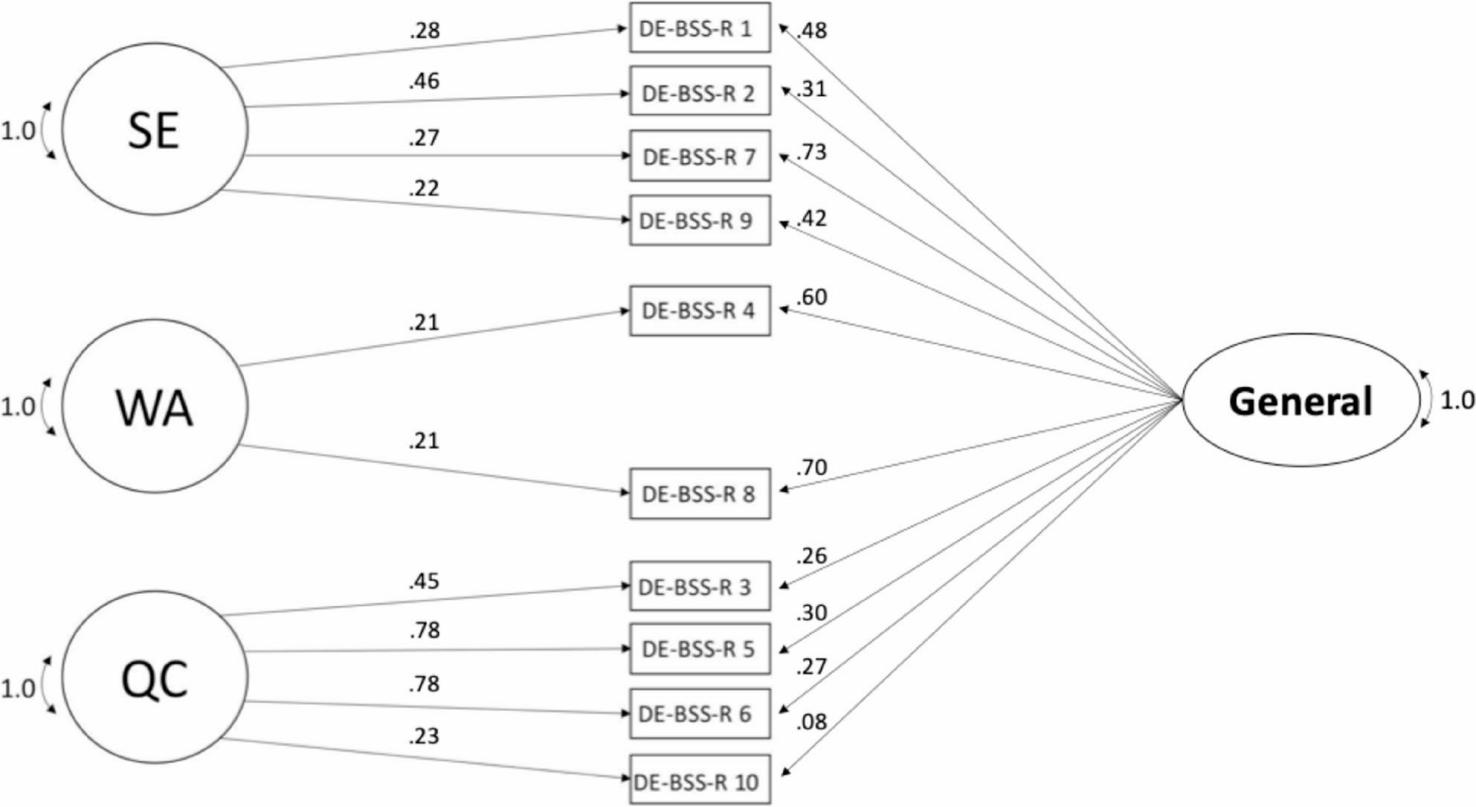
Standardized factor loadings of the bi-factor model of the DE-BSS-R

All reported results were computed using robust Maximum Likelihood estimation, although the results using Maximum Likelihood estimation were almost identical. The tri-dimensional measurement model showed excellent fit to the data with RMSEA = 0.04, CFI = 0.98, and SRMR = 0.02. The factor loadings are displayed in Fig. 1. For the bi-factor measurement model, the loadings of the two items of the WA factor had to be constrained to equality to make the model identified while the covariances are fixed to zero as they must be for an orthogonal bi-factor model (see Fig. 2). The bi-factor model also showed excellent fit to the data, with RMSEA = 0.03, CFI = 0.99, and SRMR = 0.02. However, the factor loadings of almost all items were higher in the tri-dimensional model than in the bi-factor model (for the three uncorrelated factors and the general factor). As the tri-dimensional and the bi-factor measurement models are not nested within each other, they cannot be compared by the Chi^2^ difference test. Instead, we descriptively compared fit indices, including Akaike (AIC_tri−dimensional_ = 93443.05; AIC_bi−factor_ = 93379.15) and Bayesian (BIC_tri−dimensional_ = 93648.57; BIC_bi−factor_ = 93622.03) information criteria. The bi-factor model showed slightly better RMSEA and CFI as well as lower AIC and BIC information criteria, while SRMR was identical to the tri-dimensional model.

### Multigroup CFA for educational groups

We evaluated configural, metric, and scalar invariance sequentially for the tri-dimensional measurement model of the DE-BSS-R. The configural model showed acceptable fit (RMSEA = 0.038, CFI = 0.979, SRMR = 0.027), indicating that the overall factor structure was consistent across the two groups ‘women with less than 10 years of education’ and ‘women with more than 10 years of education’. Constraining factor loadings to be equal (metric invariance) did not substantively worsen model fit (RMSEA = 0.037, CFI = 0.978, SRMR = 0.032), and neither did additionally constraining item intercepts (scalar invariance; RMSEA = 0.042, CFI = 0.968, SRMR = 0.036). These results support full scalar invariance, indicating that the DE-BSS-R measures women’s birth experience equivalently across educational groups.

### Descriptive statistics of the DE-BSS-R

The descriptive and distributional characteristics of the DE-BSS-R are displayed in Table 2. No excessive skew or kurtosis was observed, apart from item 10, which slightly exceeded the recommended cut-offs for a normal distribution [32, 33]. Additionally, 84.1% of respondents answered *strongly agree* [4] for item 10. The QC subscale was normally distributed according to skew and kurtosis but showed a similarly small range, with 26.9% of respondents having the highest possible score of 16 in this subscale.

Pearson correlation coefficients between the total score and the subscale scores can be seen in Table 3. All subscales and the total score of the DE-BSS-R were significantly and positively correlated with each other (*p* <.01). None of the correlations of the current study proved significantly different from the correlations in the original UK version (see Table 3).

**Table 2:** Descriptive and distributional characteristics of the DE-BSS-R

Item	Item content	Domain	Mean	SD	Min	Max	Skew	Kurtosis	se
DE-BSS-R 1	I came through childbirth virtually unscathed.	SE	2.79	1.15	0	4	−0.75	−3.57	0.02
DE-BSS-R 2	I thought my labour was excessively long.	SE	2.59	1.39	0	4	−0.65	−3.95	0.02
DE-BSS-R 3	The delivery room staff encouraged me to make decisions about how I wanted my birth to progress.	QC	3.06	0.99	0	4	−1.05	3.60	0.02
DE-BSS-R 4	I felt very anxious during my labour and birth.	WA	2.73	1.18	0	4	−0.82	−3.33	0.02
DE-BSS-R 5	I felt well supported by staff during my labour and birth.	QC	3.52	0.69	0	4	−1.51	5.49	0.01
DE-BSS-R 6	The staff communicated well with me during labour.	QC	3.52	0.70	0	4	−1.58	5.51	0.01
DE-BSS-R 7	I found giving birth a distressing experience.	SE	2.63	1.29	0	4	−0.64	−3.78	0.02
DE-BSS-R 8	I felt out of control during my birth experience.	WA	2.47	1.19	0	4	−0.50	−3.77	0.02
DE-BSS-R 9	I was not distressed at all during labour.	SE	1.16	0.98	0	4	0.90	3.45	0.02
DE-BSS-R 10	The delivery room was clean and hygienic.	QC	3.83	0.40	2	4	−2.25	7.27	0.01
SE	Sub-scale score (items 1, 2, 7, 9)		9.17	3.40	0	16	−0.36	−3.51	0.06
WA	Sub-scale score (items 4, 8)		5.21	2.03	0	8	−0.65	−3.23	0.03
QC	Sub-scale score (items 3, 5, 6, 10)		13.94	2.06	5	16	−1.25	4.62	0.03
Total	Total score		28.32	5.69	8	40	−0.48	−3.13	0.09

*SE* Subscale ‘Stress experienced during labour’, *WA* Subscale ‘Women’s personal attributes’, *QC* Subscale ‘Quality of care provision’, *se* standard error

**Table 3:** Correlations of DE-BSS-R subscales and total score and comparison with original UK BSS-R validation study (Hollins Martin & Martin, 2014). [18]

Scale combination	Current study *r*	UK study *r*	z	95% CI	*p*
SE – WA	0.53	0.57	−0.84	−0.13–0.06	0.40
SE – QC	0.23	0.26	−0.47	−0.15–0.10	0.64
WA – QC	0.25	0.35	−1.60	−0.21–0.02	0.11
Total score – SE	0.87	0.86	0.58	−0.02–0.05	0.56
Total score – WA	0.76	0.80	−1.49	−0.08–0.01	0.14
Total score – QC	0.59	0.63	−0.93	−0.12–0.05	0.35

*SE* Subscale ‘Stress experienced during labour’, *WA* Subscale ‘Women’s personal attributes’, *QC* Subscale ‘Quality of care provision’, *z* Fisher’s z, *95% CI* Zou’s confidence interval of the difference between correlations

### Internal consistency

Cronbach’s alpha was acceptable for the total score and just below the recommended cut-off of 0.70 for the SE and the QC subscale (see Table 4). However, alpha coefficients in the current study did not differ significantly from those in the original UK version, where these were all >0.70. The inter-item correlation of the two items of the WA subscale was in the acceptable range of 0.15–0.50 with *r* =.46. McDonalds Omega (ω = 0.76) and Omega total (ω*t* = 0.80) for the total scale were both acceptable (>0.70), while Omega hierarchical (ω*h* = 0.51) was below the recommended threshold (>0.65; 41), indicating high dimensionality [47].

**Table 4:** Cronbach’s alphas of DE-BSS-R subscales and total score and comparison with original UK BSS-R validation study (Hollins Martin & Martin, 2014). [18]

Scale	Current study α	UK study α	Χ^2^	*p*
SE	0.65	0.71	2.14	0.14
QC	0.68	0.74	2.60	0.11
Total score	0.74	0.78	2.32	0.13

*SE* Subscale ‘Stress experienced during labour’, *QC* Subscale ‘Quality of care provision’, *X*^*2*^ Chi square statistic

### Known-groups discriminant validity

Women with a non-instrumental vaginal birth differed significantly from women with an instrumental birth in their total DE-BSS-R score, *t*(1563.06) = 14.36, *p* <.001, and all three subscales: SE, *t*(1510.73) = 10.36, *p* <.001, WA, *t*(1546.45) = 15.54, *p* <.001, and QC, *t*(1515.42) = 7.22, *p* <.001. Women with a non-instrumental vaginal birth had on average a 3.12 higher total score, 95% CI [2.69, 3.54], 1.38 higher SE, 95% CI [1.12, 1.64], 1.20 higher WA, 95% CI [1.05, 1.36], and 0.59 higher QC subscale score, 95% CI [0.43, 0.75] than women with an instrumental birth. The effect sizes for the differences in the total score and the WA subscale were medium (*d* = 0.56 and *d* = 0.62, respectively), while the differences in the SE and QC subscales had a small effect size (*d* = 0.41 and *d* = 0.29, respectively).

Women with a planned caesarean section differed significantly from women with an emergency caesarean section in their total DE-BSS-R score, *t*(740) = 9.34, *p* <.001, and two subscales: SE, *t*(723) = 13.23, *p* <.001, and QC, *t*(506.49) = 3.31, *p* <.001. Women with a planned caesarean section had on average a 3.97 higher total score, 95% CI [3.13, 4.80], 3.31 higher SE, 95% CI [2.82, 3.80], and 0.58 higher QC subscale score, 95% CI [0.24, 0.93] than women with an emergency caesarean section. The effect size for the difference in the total score was medium (*d* = 0.70), large for the difference in the SE subscale (*d* = 1.0), and small for the difference in the QC subscale (*d* = 0.26).

### Divergent validity

The subscale score of WA (*r* =.01, *p* =.62) and QC (*r* =.01, *p* =.77) did not show a significant correlation with women’s age. However, the total score (*r* =.03, *p* =.036) and the subscale score of SE (*r* =.05, *p* =.003) showed very small significant correlations with women’s age.

### Convergent validity

The DE-BSS-R total score and all three subscale scores were significantly and negatively correlated with the City BiTS birth-related and general PTSD symptoms (see supplementary Table S2). Correlations between the DE-BSS-R total score and subscale scores and the birth-related symptoms were higher than the general symptoms.

### Predictive validity

All three multiple linear regression analyses were computed using Bootstrapping with 1,000 iterations due to non-normally distributed residuals and heteroscedasticity of the residuals. All three regression analyses were significant (see Table 5) and all three DE-BSS-R subscales significantly predicted the City BiTS birth-related and general PTSD symptoms as well as the EPDS total score.

**Table 5:** Multiple linear regression analyses of DE-BSS-R predicting City BiTS birth-related symptoms (Model 1), City BiTS general PTD symptoms (Model 2), and EPDS total score (Model 3)

Model	*R* ^2^	F	*p*	B	95% CI	β	t	*p*_B_
CB-PTSD	0.24	390.73	< 0.001					
SE				− 0.18	(−0.21, − 0.16)	− 0.28	−16.75	< 0.001
WA				− 0.25	(−0.29, − 0.21)	− 0.23	−13.33	< 0.001
QC				− 0.14	(−18, − 0.10)	− 0.13	−8.79	< 0.001
G-PTSD	0.05	69.08	< 0.001					
SE				− 0.08	(−0.13, − 0.04)	− 0.08	−3.99	< 0.001
WA				− 0.27	(−0.35, − 0.20)	− 0.15	−7.70	< 0.001
QC				− 0.14	(−0.20, − 0.08)	− 0.08	−4.63	< 0.001
EPDS	0.08	104.76	< 0.001					
SE				− 0.06	(−0.10, − 0.02)	− 0.06	−3.04	0.002
WA				− 0.43	(−0.50, − 0.35)	− 0.23	−12.33	< 0.001
QC				− 0.08	(−0.14, − 0.01)	− 0.04	−2.59	0.02

*SE* Subscale ‘Stress experienced during labour’, *WA* Subscale ‘Women’s personal attributes’, *QC* Subscale ‘Quality of care provision’, *CB-PTSD* City BiTS subscale birth-related PTSD symptoms, *G-PTSD* City BiTS subscale general PTSD symptoms, *EPDS* Edinburgh Postnatal Depression Scale, *p* *p*-value for the model fit, *B* unstandardized regression coefficient, *ß* standardized regression coefficient, *t* *t* statistic for *ß*, *p*_*B*_ *p*-value for *B*

*B* and its 95% CI as well as the *p*-value for *B* were computed using 1,000 Bootstrap iteration

## Discussion

The validation of the DE-BSS-R showed that it is a valid instrument to measure birth satisfaction among German postpartum women, with good psychometric properties similar to the English original [18].

Both the tri-dimensional model and the bi-factor model showed excellent fit to the data, with the bi-factor model having slightly better fit indices. However, due to the fact that bi-factor models often show statistical bias in fit indices compared to other model types [48–50], we cannot conclude with confidence that the bi-factor model is superior to the tri-dimensional model, but instead support Martin et al. [35] in the assumption that both the total score as well as the subscale scores are valid measures. The total score and the subscale scores of the DE-BSS-R were all significantly correlated with each other, and correlations were comparable to the English original of the BSS-R.

One item that stood out from the analyses was item 10 (“The delivery room was clean and hygienic.”), which had the highest mean and lowest standard deviation, the smallest range of all items, and was not normally distributed according to skew and kurtosis, with the majority of women strongly agreeing with this item and no one answering strongly disagree or disagree. Additionally, it had the lowest factor loading (< 0.3) in both the tri-dimensional and the bi-factor model. The authors of the Dutch validation of the BSS-R, who reported the same findings, state that the distributional characteristics of item 10 could contribute statistically to the low factor loading [23]. From a content point of view, the potential ceiling effect of item 10 does not come as a surprise in the context of the current study, because the hygiene standards in German hospitals (where 97.8% of the present sample gave birth, with only 2.2% of women giving birth in a birth centre or at home) are very high. Furthermore, the other three items on the QC subscale were also rated very highly, resulting in a high QC subscale mean score and indicating a great level of satisfaction among the participating women with the overall quality of care they received during labour and birth.

To the best of our knowledge, we were the first to demonstrate that the BSS-R assesses women’s birth experience equivalently across educational groups, specifically among women with 10 years or less and more than 10 years of education. This finding indicates that the instrument functions consistently across these groups, allowing for meaningful comparisons of latent means. Establishing measurement invariance is essential to ensure that observed differences between groups are not due to measurement bias. In the context of perinatal research and policy, this contributes to more equitable assessment and understanding of how birth experiences may differ by socioeconomic background. As the association between women’s education and birth experience is still unclear [7], this finding importantly demonstrates that the DE-BSS-R can be used as a reliable instrument to further study the relationship between these two constructs.

Both Cronbach’s alpha and Omega indicated acceptable internal consistency of the total score, which is used more often than the subscale scores in clinical practice and supports its reliability. Internal consistency for the SE and QC subscales was just below the recommended cut-off but did not significantly differ from the acceptable values in the original UK version, indicating that subscale scores can be used in research alongside the total score [35].

When testing the DE-BSS-R for known-groups discriminant validity, almost all hypotheses could be confirmed. Women with a non-instrumental vaginal birth rated their overall satisfaction with birth as well as all three subscale domains better than women with an instrumental birth (instrumental vaginal birth, planned caesarean, emergency caesarean), which mostly mirrors findings from other BSS-R validations [20, 51, 52]. We did not expect a significant difference between the birth modes in the QC subscale, but the effect size for this comparison was small. Considering that instrumental births usually involve more emergency situations, in which the life of mother or baby may be in danger and obstetric staff must act quickly, a negative effect on the perceived communication with staff and involvement in decision making processes may be an understandable outcome but nevertheless indicates a need for improvement. Moreover, we confirmed the finding of earlier validations [19, 22, 23] that the BSS-R can distinguish between planned and emergency caesarean sections: women with an emergency caesarean section showed lower total and SE and QC subscale ratings of the DE-BSS-R. As mentioned above, it is natural that emergency situations are perceived as highly stressful and with most women being under general anaesthesia for an emergency caesarean section, this might leave less room for positive interactions with staff. However, it is unclear why there was no difference in the WA subscale, which measures women’s anxiousness and feeling of control during labour and birth. To the best of our knowledge, this has so far only been reported by the authors of the Dutch validation study [23].

Divergent validity could be shown without issues for the WA and QC subscales, as these did not correlate significantly with women’s age. The total score and the SE subscale score showed very small significant correlations. However, this is most likely due to the very large sample size of the current study, which increases the power to find even the smallest effects, which are different from zero but do not have any meaning in terms of content.

Convergent and predictive validity could be confirmed by showing significant correlation and/or regression coefficients between the DE-BSS-R subscales and the City BiTS subscales CB-PTSD and general PTSD as well as depression symptoms. These findings highlight the importance of birth satisfaction for women’s postpartum mental health, especially CB-PTSD symptoms, which showed the strongest association with birth satisfaction. The latter finding also suggests that birth satisfaction is not only associated with generic PTSD symptoms due to another event than childbirth, which could have existed prior to the recent birth and are diagnosed by chance when screening for PTSD symptoms after birth, but that a more negative birth experience seems to predict PTSD symptoms which are directly related to traumatic birth events [22]. However, we tested this with cross-sectional data and future studies should confirm these findings with longitudinal data. If studies have longitudinal data, they should also attempt to test the test-retest reliability of the BSS-R, which we were not able to do with our cross-sectional dataset, but which has been successfully done in previous validations [22].

Further limitations of the current study are the location of recruitment, the homogeneity of the sample, and the time range of when women completed the telephone interview. We interviewed only women who gave birth in Dresden, a city in Eastern Germany, and therefore it is unclear if the current findings can be generalized to the whole of Germany. For instance, intervention rates (e.g., caesarean section rates) are typically lower in Dresden compared to the rest of Germany (caesarean section rate in Dresden 22.1% [53] vs. 31.9% in Germany [54]). Additionally, the majority of women in our sample had a moderate to high income and was in a stable partnership, which might make our findings non-generalizable to less privileged women. Moreover, women completed the BSS-R between one week and one year postpartum, which could have impacted the results. However, the majority completed the DE-BSS-R 13.2 weeks postpartum and Emmens et al. [23] and Nakić Radoš et al. [19] showed that time since birth has no significant association with BSS-R scores, supporting the assumption that birth satisfaction is rather stable over time.

The current study also has several strengths. We were able to show in a very large sample of postpartum women that the DE-BSS-R is a valid instrument to measure birth satisfaction in Germany. Previously, there were only three validated instruments available in German, which measure birth experience [55]: the Wijma Delivery Experience Questionnaire with 33 items [56], Salmon’s Item List with 20 items [57], and the Childbirth Experience Questionnaire with 25 items, which is only suitable for first-time mothers [58]. Due to their length, none of these instruments are suitable for short surveys or use in clinical practice, making the DE-BSS-R a well-suited alternative with only 10 items. Its availability in several languages also holds the possibility to offer it to non-German speaking women in German hospitals and use it in international comparison studies of birth satisfaction like the INTERSECT study [59], facilitating cultural comparisons and comparisons between different contexts of maternity care. Finally, to the best of our knowledge, this is the first validation of the BSS-R, which used telephone interviews instead of paper-pencil or online questionnaires. As the psychometric properties of the DE-BSS-R were good and, in most cases, similar to the original English version and other translations, we assume that there are no differences between these modes of assessment for the BSS-R.

## Conclusion

In conclusion, the German cross-cultural adaptation of the BSS-R showed good psychometric properties and discriminant, divergent, convergent, as well as predictive validity. Both the total score and the three subscale scores are valid to quickly measure German women’s satisfaction with birth in research and clinical practice.

## Supplementary Information

Supplementary Material 1.

## Data Availability

The datasets generated and/or analysed during the current study are not publicly available due to legal and ethical constraints as public sharing of participant data was not included in the informed consent of the study, but are available from the corresponding author on reasonable request.

## Abbreviations

BSS-R: Birth Satisfaction Scale-Revised
CB-PTSD: Childbirth-related posttraumatic stress disorder
CFA: Confirmatory factor analysis
CFI: Comparative fit index
City BiTS: City Birth Trauma Scale
DE-BSS-R: German version of the Birth Satisfaction Scale-Revised
EPDS: Edinburgh Postnatal Depression Scale
G-PTSD: General posttraumatic stress disorder symptom subscale of the City Birth Trauma Scale
ICHOM: International consortium for health outcomes measurement
INTERSECT: International Survey on Childbirth-Related Trauma
INVITE: INtimate partner VIolence Treatment prEferences
QC: Quality of care provision (BSS-R subscale)
RMSEA: Root mean squared error of approximation
SE: Stress experienced during labour (BSS-R subscale)
SRMR: Square root mean residual
WA: Women’s personal attributes (BSS-R subscale)
