# Supplementary material for: Cross-cultural adaptation and validation of the German version of the Birth Satisfaction Scale-Revised (BSS-R)
Source: BMC Public Health. 2025 Nov 22;25:4280. doi: 10.1186/s12889-025-25563-2 (PMC12723901; doi:10.1186/s12889-025-25563-2)
Supplement: Supplementary file 1 — Supplementary Material 1. [file 12889_2025_25563_MOESM1_ESM.docx]

**Supplemental materials**

**Table S1**

*Items of the original UK version BSS-R and the DE-BSS-R*

| **Subscale** | **UK version BSS-R** | **DE-BSS-R** |
| --- | --- | --- |
| SE | I came through childbirth virtually unscathed. | Ich bin nahezu unversehrt durch meine Wehen und die Geburt gekommen. |
|  | I thought my labour was excessively long. | Ich dachte, meine Wehen und die Geburt waren übermäßig lang. |
|  | I found giving birth a distressing experience. | Ich empfand die Geburt als eine belastende Erfahrung. |
|  | I was not distressed at all during labour. | Ich fühlte mich während meiner Wehen überhaupt nicht belastet. |
| WA | I felt very anxious during my labour and birth. | Ich fühlte mich während meiner Wehen und der Geburt sehr ängstlich. |
|  | I felt out of control during my birth experience. | Ich hatte während meiner Geburtserfahrung das Gefühl, keine Kontrolle zu haben. |
| QC | The delivery room staff encouraged me to make decisions about how I wanted my birth to progress. | Das Personal im Kreißsaal ermutigte mich zu entscheiden, wie ich die Geburt voranschreiten lassen wollte. |
|  | I felt well supported by staff during my labour and birth. | Ich fühlte mich während meiner Wehen und der Geburt gut vom Personal unterstützt. |
|  | The staff communicated well with me during labour. | Das Personal hat während meiner Wehen und der Geburt gut mit mir kommuniziert. |
|  | The delivery room was clean and hygienic. | Der Kreißsaal war sauber und hygienisch. |

*Note*. Participants have the following answer options for all 10 items: *Strongly disagree* (German: *Stimme überhaupt nicht zu*), *disagree* (German: *Stimme nicht zu*), *neither agree nor disagree* (German: *Stimme weder zu noch nicht zu*), *agree* (German: *Stimme zu*), *strongly agree* (German: *Stimme voll und ganz zu*).

**Table S2**

*Spearman’s rho correlation coefficients between DE-BSS-R total score and subscale scores and City BiTS birth-related and general PTSD symptoms*

|  | **1.** | **2.** | **3.** | **4.** | **5.** | **6.** |
| --- | --- | --- | --- | --- | --- | --- |
| **1. DE-BSS-R total score** | 1 |  |  |  |  |  |
| **2. SE** | .87 | 1 |  |  |  |  |
| **3. WA** | .76 | .53 | 1 |  |  |  |
| **4. QC** | .55 | .22 | .25 | 1 |  |  |
| **5. City BiTS CB-PTSD** | -.46 | -.42 | -.38 | -.20 | 1 |  |
| **6. City BiTS general PTSD** | -.21 | -.16 | -.19 | -.14 | .28 | 1 |

*Note*. SE = Subscale ‘Stress experienced during labour’; WA = Subscale ‘Women’s personal attributes’; QC = Subscale ‘Quality of care provision’; City BiTS CB-PTSD = City BiTS subscale of birth-related PTSD symptoms; City BiTS general PTSD = City BiTS subscale of general PTSD symptoms.

All correlations *p* < .001.
